# Smartphone as a Sensor in mHealth: Narrative Overview, SWOT Analysis, and Proposal of Mobile Biomarkers

**DOI:** 10.3390/s25123655

**Published:** 2025-06-11

**Authors:** Alessio Antonini, Serhan Coşar, Iman Naja, Muhammad Salman Haleem, Jamie Hugo Macdonald, Paquale Innominato, Giacinto Barresi

**Affiliations:** 1 Knowledge Media Institute, The Open University, Milton Keynes MK7 6AA, UK; iman.naja@open.ac.uk; 2 School of Engineering & Innovation, The Open University, Milton Keynes MK7 6AA, UK; sehran.cosar@open.ac.uk; 3 School of Electronic Engineering and Computer Science, Queen Mary University, London E1 4NS, UK; m.haleem@qmul.ac.uk; 4 School of Psychology and Sport Science, Bangor University, Bangor LL57 2DG, UK; j.h.macdonald@bangor.ac.uk; 5 Oncology Department, Betsi Cadwaladr University Health Board, Bangor LL57 2PW, UK; pasquale.innominato@wales.nhs.uk; 6 Warwick Medical School & Cancer Research Centre, University of Warwick, Coventry CV4 7AL, UK; 7 Bristol Robotics Laboratory, University of the West of England, Bristol BS16 1QY, UK

**Keywords:** digital health, mHealth, smartphone, passive monitoring

## Abstract

Digital applications for supporting health management often fail to achieve large-scale adoption. Costs related to purchasing, maintaining, and using medical or sensor devices, such as smartwatches, currently hinder uptake and sustained engagement, particularly in the prevention and monitoring of lifelong conditions. As an alternative, smartphone-based passive monitoring could provide a viable strategy for lifelong use, removing hardware-related costs and exploiting the synergies between mobile health (mHealth) and ambient assisted living (AAL). However, smartphone sensor toolkits are not designed for diagnostic purposes, and their quality varies depending on the model, maker, and generation. This narrative overview of recent reviews (narrative meta-review) on the current state of smartphone-based passive monitoring highlights the strengths, weaknesses, opportunities, and threats (SWOT analysis) of this approach, which pervasively encompasses digital health, mHealth, and AAL. The results are then consolidated into a newly defined concept of a *mobile biomarker*, that is, a general model of medical indices for diagnostic tasks that can be computed using smartphone sensors and capabilities.

## 1. Introduction

Mobile digital health applications (mHealth) remain a niche, despite being widely recognised as a critical component of the sustainable future of healthcare systems, especially considering contexts such as remote care and ambient assisted living (AAL) [1,2,3]. The pressures of an ageing population and increased life expectancy demand enhanced disease prevention strategies and better support for people living with chronic conditions to self-manage their care. Encouragingly, a staggering number of digital health solutions have been produced since the introduction of smartphones. However, despite their abundance, their uptake is confined to fitness and consumer well-being applications. They are rarely used in medical device–grade solutions and have not been adopted at scale in public health or prevention interventions, healthcare service delivery, or self-management of chronic conditions. Uptake and sustained engagement in digital health remain well-known issues, even in the context of clinical studies [4,5], which arguably take place under more favourable conditions than market-led adoption at scale. Furthermore, the fast development of Artificial Intelligence (AI) technologies [6] highlights the need to review current approaches in light of the new opportunities offered by dedicated AI chipsets and services.

Smartphone-only passive monitoring leverages the multiple sensors embedded in smartphones to track many aspects of users’ activities. Potentially, this data, supplemented with the addition of further sensors, could be used to monitor health status with no extra effort required from users (as data collection is carried out in the background) and no extra costs for healthcare systems (as smartphones are patient-owned). Passive monitoring is particularly promising for population-scale prevention campaigns and the management of chronic conditions, as it enhances uptake and sustained engagement by reducing the user burden associated with the active input required by traditional monitoring approaches. However, smartphone-based passive monitoring has yet to become a mainstream approach in market-available digital health solutions, so its viability beyond proof-of-concept and pilot studies is not clear [7].

To this end, this contribution is a critical narrative overview based on recent reviews (a narrative meta-review), with the aim of assimilating the known strengths, weaknesses, opportunities, and threats (SWOT) [8] of smartphone-based passive monitoring that have emerged over the past decade. The results of the SWOT analysis are then used to rethink a general architecture—informed by the current trends in AI and edge computing—to develop a new concept, i.e., *mobile biomarkers*.

This narrative meta-review addresses the following three questions:

Q1 What is the status of smartphone-only passive monitoring as an approach?
Q2 What advancements are needed to make this approach more reliable and adopted more widely?
Q3 How could the latest advancements in AI benefit mobile-based passive monitoring?

Smartphone-only passive monitoring presents challenges and limitations that are both specific to certain applications and general to the approach. This contribution focuses on the latter. In this spirit, we scanned reviews on passive monitoring, looking for findings that cut across conditions or specific techniques, which we then organised into a SWOT analysis.

**Figure 1:**
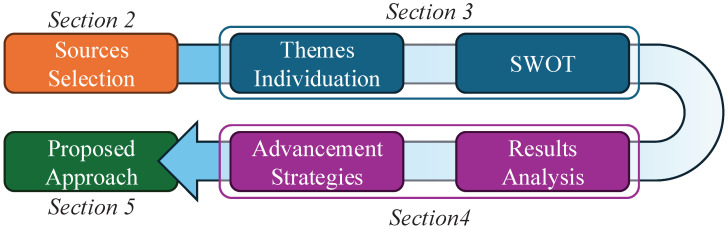
Study approach.

The structure of the remaining sections of this paper is shown in Figure 1. Firstly, the selection of review papers, presented in Section 2, provides a broad overview of the focus of such studies. This section also clarifies the rationale behind our choice of a narrative meta-review and why a SWOT analysis is an appropriate method for addressing our research questions. The SWOT analysis is presented in Section 3. In this section, we identify recurrent themes and then cluster and analyse them using the SWOT framework. The results of the SWOT analysis are then discussed in Section 4. This discussion addresses the three above questions and also outlines two strategies to address the identified weaknesses and threats of the approach to passive monitoring.

Finally, Section 5 outlines a potential general approach that incorporates the strategies for improvement identified in earlier sections. The proposed approach extends the concept of digital biomarkers [9] to leverage the unique smartphone capabilities for ubiquitous computing. Accordingly, we propose a class of medical indices that could be computed by multimodal analyses of the data taken from smartphone sensors, which could then be tailored to specific diagnostic tasks; we label these indices *mobile biomarkers* (MBs). We propose a modular architecture addressing the issues emerging from the narrative meta-review, such as device storage degradation and privacy risks involved in third-party cloud providers. The MB architecture is defined as a general framework for deploying interoperable, mutually interchangeable, and modular biomarkers on personal devices to facilitate future research. From a technical perspective, the MB architecture incorporates current trends in edge AI (running on new smartphone neural network–dedicated chips)—an untapped potential in digital health applications—and federated machine learning architectures.

## 2. Methodology

Our analysis aims to understand the potential benefits and drawbacks of smartphone-only passive monitoring in the context of lifelong use. In this light, we approached this investigation as a narrative *meta-review*—a synthesis based on our goal and qualitative constraints [10]—to preliminarily explore the following topics: (1) identifying the characteristics that are in the way of large-scale adoption, and (2) finding a general solution to further the development of lifelong smartphone-based monitoring.

We chose not to utilise a systematic review for several reasons. First of all, a narrative meta-review offers higher flexibility in both scope and approach, making it more suitable for an exploratory and critical discussion of emerging approaches. Advances in mobile technologies are increasing year by year. Thus, this rapidly evolving scenario could be advantageously described through a narrative review (a meta-review, in this case) rather than a systematic one, which could be too restrictive to incorporate sources and trends that may be crucial for highlighting insights and innovations in fast-moving domains such as mHealth. Consequently, a narrative meta-review can serve as a valuable step toward guiding future research and development, especially if it is structured with industrial stakeholder-friendly tools like a SWOT analysis. Furthermore, by narratively referring to material already published in reviews proposed by different teams of authors, we can consider an unbiased selection of sources, but still discuss the advantages and disadvantages of smartphone sensors.

The analysis progresses in two steps. First, we define our position in the form of hypotheses and constraints. Next, we identify the dominant themes related to our research questions and critically analyse them accordingly.

For the reasons outlined in the introduction, and without adhering to a systematic approach, which is not required for this preliminary narrative meta-review, we searched (on Scopus, Google Scholar, IEEE Xplore, and ACM Library) for reviews using a combination of keywords such as “smartphone sensors”, “health”, and “passive monitoring” while excluding terms like “wearables” and “mobile” as these may also refer to other personal devices and combinations of smartphones and sensors. We chose to focus on reviews, given the broad scope of our inquiry into whether smartphone-only passive monitoring can serve as a useful approach for prevention and lifelong monitoring in general. Reviews offer a broader perspective that aligns more closely with our research questions than specialist papers focused on specific conditions or treatments.

### Selection of Reviews

Reviews were identified by searching common repositories like Scopus and Google Scholar. The search used the intersection of keywords “review”, “smartphone”, and “digital health”, and was then refined by including “monitoring”. From the initial results, we excluded all reviews that focused exclusively on user-reported data and apps for active user assessments (such as cognitive tests) or contributions that focused solely on external sensors (like wearables or smart home devices) or used smartphones simply as a communication bridge to collect and send data to the cloud.

The selection process identified 14 reviews. Preliminary analysis of selected reviews highlighted clear emerging themes cutting across the reviews: (1) mental, well-being, or physical health; and (2) technical or clinical results. Table 1 shows how most reviews focused on different aspects of mental health by choice or because this was the focus of early applications [11].

Notably, reviews spanned almost a decade, demonstrating the recurring interest and expectations for smartphone-based passive monitoring and the continuous efforts in testing specific applications across medical fields. Next, Section 3 presents an analysis of the themes emerging from the reviews, organised according to the macro-categories of a SWOT analysis.

## 3. SWOT Analysis

The SWOT analysis is a versatile method that applies very well to medical devices, efficiently informing a diverse range of stakeholders through concrete, insightful, and actionable findings [8]. A SWOT analysis offers a helpful overview that can engage researchers, clinicians, managers, policymakers, and end users in the improvement and subsequent adoption of a novel tool or service. Using SWOT analyses in the literature and project proposals also helps reduce the gap between academia and stakeholders, especially in contexts of technology research [23,24].

In general, a SWOT analysis identifies the strengths, weaknesses, opportunities, and threats relevant to activities like strategic planning and decision-making (in fields like marketing, for instance) by identifying competitive advantages and mitigating potential issues while holistically pondering contextual factors. In particular, a SWOT analysis classifies positive and negative aspects of its target (e.g., any asset, from a product to a process) in terms of its internal (within the reference system—e.g., the context of use) or external (within a wider system—e.g., the society as a whole) origin. Consequently, favourable features can be internal (strengths, elements of the asset that can offer an advantage) or external (opportunities, elements beyond the asset that can increase its impact). The same distinction occurs between internal (weaknesses, disadvantageous elements characterising the asset) and external (threats, potentially troublesome elements beyond the asset) aspects that may hinder strategies based on a certain asset.

The distinctive feature of our work is its focus on the positive healthcare applications of smart monitoring. While a few other SWOT analyses have examined smartphone-based passive monitoring in general terms or in other domains, ours is, to the best of our knowledge, the first to provide an overview of the use of smartphone data in mHealth (other works consider smartphones as a cloud bridge or interface for external medical devices) for diagnostic and prognostic goals. Our decision to use a SWOT analysis was made after considering several different methodologies with a similar scope [25]. However, some of them seem too related to internal factors (e.g., SOAR [26] and NOISE [27]), too close to strategic choices that can be conducted in actual business plans (e.g., SCORE [28]), or too focused on the analysis of competitors (e.g., Five Forces [29]). Consequently, we considered the SWOT analysis—with its clear presentation of internal and external factors as positive or negative—as the most balanced and appropriate tool for introducing a holistic discussion on evolving technological innovations, encompassing perspectives moving between academia and industry (as demonstrated in similar studies in the literature) [23].

**Figure 2:**
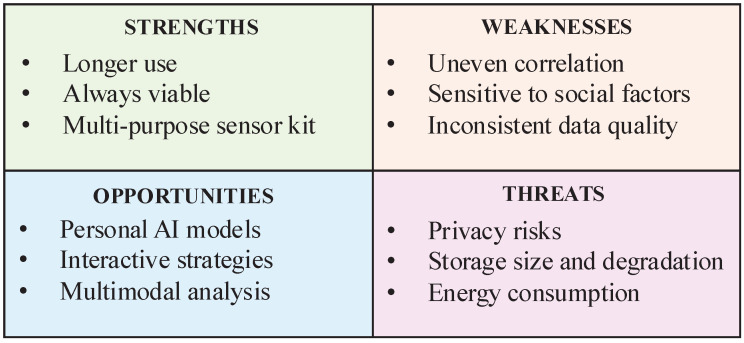
The analysis of findings highlights the associations between strengths, weaknesses, opportunities, and threats of passive monitoring.

The analysis evidenced a clear pattern concerning the stage of development of this approach, which, notably, did not change across both earlier and later reviews, as summarised in Figure 2. In the following sections, we discuss our findings, starting with strengths, which emerged as the most consistent yet least addressed themes, followed by threats of under-explored implementation issues, weaknesses strongly grounded in clinical studies, and opportunities, highlighting insights from the different perspectives of reviewers.

### 3.1. Strengths

Passive monitoring has been widely recognised as a viable approach to providing “objective data about the recovery trends” [21], offering more precise and in-depth insights “without imposing burdens on participants” [22].

Another core strength is the adaptability of the smartphone sensor kit, which allows for a wide range of studies. Notable applications of passive monitoring include the following:

- Health diagnosis [17], including methods based on human activity recognition [20].
- Mental health monitoring [7,13,15,19], specifically of loneliness and social isolation [18,22,22].
- Fitness and physical well-being [13,14].
- Specific application to cancer monitoring, tackling a wide range of cancer symptoms ranging from physical to mental health [21].

The strategy’s viability was never questioned; instead, “collected phone features showed significantly better performance than traditional demographic measures” [22]. A review on monitoring cancer patients highlighted two studies reporting that smartphones outperformed bracelet-wearing time (87% against 57%) [30,31] and suggested that “age might not pose a barrier” to the adoption of passive monitoring.

### 3.2. Threats

Beyond evidence of efficacy, the failure of passive sensing can stem from barriers to adoption by caregivers and patients, or from new regulations and policies related to privacy and data protection. Concerning the latter, changes in regulation and technology providers’ policies threaten data collection with an impact that “is not predictable” [11]. Pilot studies did not specifically embed these aspects in their study designs but focused exclusively on participants and caregivers, leaving issues related to policies unaddressed.

Pilot studies showcased good responses from patients, at least at the clinical study level. A review of studies on healthcare professionals’ perspectives evidenced the need for addressing “risk management and data security procedures” [19]. Indeed, one review highlighted how issues of privacy and data protection are not being accounted for in studies [18] and that its acceptability by patients is debatable [15,18].

Smartphone storage and energy consumption are critical threats to the viability of passive monitoring in its current iteration. The fast degradation of battery life due to continuous data collection is a major concern for users [18,22] as well as data storage and transmission costs [32]. These issues of storage and energy will impact the granularity of data, and require a trade-off “dilemma” between cloud and transmission expenses, energy, and privacy [16,22]. Furthermore, this approach may push the hardware beyond its intended uses, potentially leading to unforeseen failures like data corruption “caused by continuously writing data from the sensors and the finite number of read-write cycles of the flash memory” [33]. Additional aspects that should be considered beyond the scope of this overview can be found in the problematic use of the smartphone itself, as in [34].

### 3.3. Weaknesses

Case studies have shown comparable weaknesses related to the inconsistency of results. For instance, in activity recognition approaches, “there was little evidence that algorithms trained using data collected in these controlled settings [lab] could be generalised to free-living conditions” and “no single activity recognition procedure was found to work in all settings” [20]. Mental health is also an equivalent domain rigged with inconsistent findings “potentially influenced by demographics and personalities” [15], weak results, e.g., limited to the “comparisons between at-risk and normal populations or correlations with questions generating explicit self-identification as being lonely” [15], and no clinical value [22] due to the lack of interventional studies [13].

The unreliability of results is a strongly perceived issue that could potentially impact the therapeutic relationship [19]. One suggested approach calls for proactive intervention by curating lists of verified apps [12].

A recognised root cause of the unreliability of passive monitoring is the inconsistency of data quality [16,20], which could be addressed through medical device certification [12]. Limits or the absence of clinical studies also contribute to the current uncertainty of results. Studies often involve very small cohorts with short follow-up periods [7], disregarding basic notions like the distinction between loneliness and social isolation, or key demographic characteristics [18].

### 3.4. Opportunities

As an approach, passive monitoring has room for improvement. It is worth considering that the “deliberate exclusion of active sensing based on this assumption limits our understanding of potential insights that active sensing approaches can offer” [15]. A solution could come from considering an interactive approach that could also leverage more smartphone-based computing, particularly to develop personal models [13] trained with the help of users [20].

Another significant opportunity emerges from the “significance of multimodality compared to unimodality in most cases” [15]. This opportunity also lies in the ability to bring context into consideration—thanks, again, to the variety of sensors ranging from physical to social and behavioural (such as GPS, Bluetooth proximity, and call durations) [22]—that leads to “outstanding efficacy by incorporating both temporal and contextual interactions within and across modalities” [15].

## 4. Discussion

In response to Q1 on the status of smartphone-only passive monitoring, the analysis revealed that the major *threats* lie in its under-explored issues concerning its *implementation* beyond the remit of pilot studies. *Weaknesses* emerge instead on the *precision* of the relaxed constraints of passive monitoring (which is also a source of its *strengths*). Indeed, not involving strict user input protocols and the use of non–medical device-grade sensors are the cornerstones of the proven *adaptability* of the approach in terms of conditions of use and objectives. More interestingly, the analysis highlights how the approach itself remains not fully mature as an idea, with several promising *opportunities*—yet to be incorporated into a coherent vision—around *data* collection and processing.

**Figure 3:**
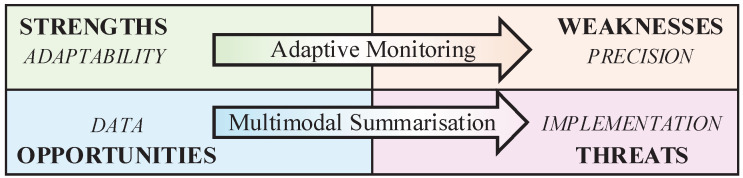
The analysis evidenced strong connections between weaknesses and the precision limitations of passive monitoring, threats and implementation challenges, opportunities and advances in multimodal and data analysis, and strengths and the adaptability of smartphone sensor kits.

Concerning Q2 on necessary advancements, data privacy and storage threats must be addressed directly with a solution that enables consistently better and more reliable results. One possible solution lies in multi-sensor analysis, which could eliminate the need to store individual sensor data (either on devices or in the cloud). This solution involves multimodal summarisation, which combines multi-sensor analysis with the selective retention of only those data vectors that are significant for a given monitoring task. Similarly, weaknesses related to precision should be addressed by building solutions based on the adaptability of smartphones. Specifically, smartphones offer a unique opportunity to implement a hybrid approach that complements passive monitoring with ad hoc user engagement to fill data gaps and ground the analysis in user insights (adaptive monitoring). What emerges is a two-fold strategy (summarised in Figure 3) that uses strengths and opportunities to overcome the evident weaknesses and threats to smartphone-based passive monitoring.

A strategy to further advance passive monitoring should connect the sensors—in a broader sense of physical and software ones, also derived from multimodal analysis—with specific diagnostic, predictive, and prognostic tasks, i.e., to convey actionable information about an individual’s bodily function [35]. On the one hand, the issue of precision could be addressed through a targeted analysis for each condition (as emerged from the meta-review). On the other hand, the solution should offer general value and be capable of addressing the growing variety of analytic approaches and diagnostic tasks, while also decoupling lifelong monitoring from any specific provider. As research advances or personal needs evolve, users (guided by caregivers when appropriate) should be able to customise monitoring according to their individual needs and goals. A general approach also supports two important decouplings:

- Decoupling advancements in analytic techniques from the implementation and deployment of virtual sensors.
- Decoupling data collection from third-party providers (private or public), enabling the creation of a personal data vault for individual use or for sharing with public or private care providers as needed throughout one’s lifetime.

The closest approach to a solution that we identified is based on the concept of digital biomarkers. In the following, we explore how digital biomarkers could be expanded to incorporate the specific characteristics of smartphone-based passive monitoring.

The last question, Q3, concerning the benefits of AI advancements to the passive monitoring approach, is addressed by looking at the following:

1. How AI technologies are changing user interaction modalities, replacing traditional forms and wizards with conversational interfaces [36].
2. How distributed architectures can enable decentralised training of AI, eliminating the need to transfer data, thereby reducing privacy risks while distributing the computational burden to the edge [37,38,39].

The last point will be addressed in the next section, where model-based and federated machine learning are used as potential technological approaches for deploying and integrating a distributed network of personalised models. Concerning AI in user interaction, it is worth noting the significant potential of large language models—and generative AI in general—in embedding and adapting rigid content like Q&A, manuals, and questionnaires. This conversation-based paradigm could solve the challenges of self-screening by adapting to users’ evolving goals and, overall, enhancing the effectiveness of support content by shifting the burden of finding, selecting, interpreting, and tailoring general guidelines to the users’ specific needs. While the reliability of generative AI is still an open issue, great progress is being made using multi-agent patterns, pairing agent generators with controllers specialised in identifying sub-par or wrong outputs. Notably, progress has also been made in small open-source models that could, in theory, be fine-tuned and run on personal devices, while being connected to larger remote resource pools to find, retrieve, and adapt relevant recommendations.

Considering the outcome of our SWOT analysis and the responses to the aforementioned questions structuring this overview, it is advantageous (for the reader and the scientific-technological community) to propose a potential approach to take advantage of the mobile innovations in health sensing. The next section will introduce an example of an approach that specifically employs the tools offered by AI systems and the features of mobile devices.

## 5. Proposed Approach—Mobile Biomarkers

Biomarkers are measurable medical indices that represent healthy or pathological conditions, enabling interventions in predictive and personalised medicine. “Digital biomarkers” [40,41] are biomarkers generated by digital health technologies, e.g., systems based on sensors, software, computing platforms, and connectivity features for healthcare applications (division of All Hazards Response, Science, and Strategic Partnerships, Office of Strategic Partnerships and Technology Innovation, Center for Devices and Radiological Health, U.S. Food and Drug Administration, Silver Spring, MD, USA) [42]. Digital biomarkers are obtained through the interaction between a user and a digital system, potentially even if a device is not directly used for medical reasons. Such an approach can feed a wide range of advanced (even experimental) assets in precision medicine, from the “omics” to digital twins [43,44].

More than any other digital device, smartphones are always—if not worn—close to users and are potentially capable of capturing contextual and activity-related data that can significantly enhance monitoring quality. In this view, mobile biomarkers are medical indices resulting from the interaction with, specifically, a smartphone-like device ubiquitous in user activities and with onboard sensing and computational capabilities. From a technical perspective, a mobile biomarker functions as a virtual sensor that integrates physical sensor data with contextual and activity information through multimodal analysis, guided by a diagnostic, predictive, or prognostic task to generate a medical index. As a virtual sensor, a mobile biomarker should produce a new summarised data stream—replacing the need to keep raw sensor data and reducing privacy risks—optimised for pre-allocated storage space and clinical significance. In this summarised format, the data is substantially reduced and well-suited to the limited storage and energy demands required for transmission.

**Figure 4:**
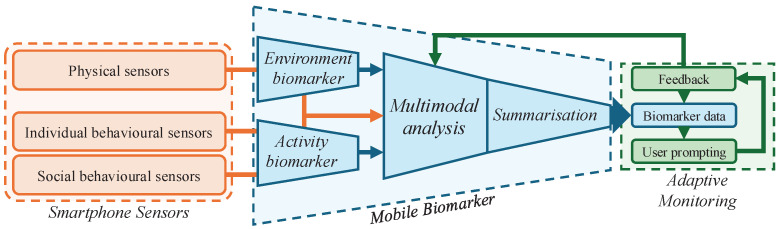
The general architecture of a mobile biomarker is compositional and should include environmental and activity analysis, bespoke multimodal analysis of smartphone sensors, and summarisation. Biomarker data are then embedded into an interactive monitoring system, which prompts users to ground or correct data when necessary and improve the analysis through a user feedback loop.

Figure 4 presents a general architecture for a mobile biomarker, which is compositional and includes biomarkers for environmental and activity-based contextual analysis. The architecture also includes a summarisation module and a feedback channel from the adaptive monitoring. In the following, we provide recommendations for the implementation of mobile biomarkers.

### 5.1. Multimodal Summarisation

The concept of multimodal data is associated with different data types (like image, text, and time series), which need to be linked together for modelling purposes via appropriate data fusion techniques [45]. Due to communication, energy, and storage constraints, edge computing requires data reduction schemes. Traditionally, raw data, without consideration of its unique properties, is treated generically and compressed using predefined transformations [46] or deep learning-based autoencoders [47]. Unlike these approaches, mobile markers exploit the properties of the data.

Some efforts have focused on designing and developing a multimodal approach that uses digital biomarkers (such as heart rate variability, eye tracking, and voice features) for childhood mental health screening [48]. Another study utilised smartphone digital biomarkers to estimate gait and balance to manage Parkinson’s disease [49]. The authors collected signals such as accelerometer data, from which gait-related variables (e.g., step series, position, acceleration) and balance-related variables (e.g., tremor frequency) were calculated. Moreover, voice recordings were used to extract temporal, frequency, and amplitude features, and mobile tapping data were recorded for directional perception. Questionnaires via a mobile app based on demographics were utilised to understand their impact on the aforementioned signals. They utilised traditional machine learning methods such as random forest to achieve an accuracy of up to 77% when combining all tasks. A multimodal deep neural network-based attention mechanism was developed to diagnose multiple sclerosis via smartphone data [50]. The authors utilised a mood questionnaire, symbol mapping, walking, balance assessments, and other measures, which were later linked together, followed by an attentive attention mechanism, with an accuracy of 88%.

A mobile biomarker encodes a type of analysis for a specific diagnostic task, which guides the relevance of data and what, from the analysis, is worth being considered and, therefore, stored away. From a technical perspective, analysis, summarisation and compression can be combined to identify anomalies and relevant events among large quantities of data [51], such as multiple streams of smartphone sensors.

Clinical research produces new techniques, presenting both the opportunity and the challenge of providing access to the latest state-of-the-art technology. Similarly, users’ needs evolve over time, whether across the lifespan or through different stages of treatment, monitoring, and recovery. In this context, mobile biomarkers should offer a standardised description—an interface for analysis—that specifies which sensor data should be considered, which algorithms or models should be applied, and how the results should be structured to support their intended use.

From a technical perspective, a mobile biomarker should be a software interface that is agnostic to specific languages and platforms, possibly described using standards like HL7’s Fast Healthcare Interoperability Resources (FHIR) [52] and interoperability approaches like model-based machine learning [53]. Describing multimodal analysis using medical standards would enable the integration of biomarker results into healthcare information systems. Model-based machine learning would instead facilitate the rapid development of biomarkers by including mode descriptions as part of the biomarker and embedding model code within the deployment architecture (instantiating the model description on the target platform). An alternative strategy is federated machine learning [54], which allows the deployment of pre-trained models and the sharing of training and results across devices without compromising privacy. The increasing presence of neural processing units in smartphones [55] enables the use of sophisticated techniques and supports the execution of recent, computationally demanding AI models. This aligns with our idea of multimodal summarisation, which embeds models and biomarkers.

### 5.2. Adaptive Monitoring

The concept of mobile biomarkers brings multimodal summarisation solutions to life as a declarative component—an interface that can be implemented and deployed as necessary on different types of hardware and platforms. The second part of the solution is adaptive monitoring, which combines into one strategy an *interactive machine learning* (IML) approach with timely, on-demand active user engagement.

IML is a class of solutions designed to address performance bottlenecks caused by not including users in the loop [56]. IML particularly fits the technical constraints of running mobile biomarkers on smartphones, accommodating variability in usage, available data, and the opportunities presented by context analysis. Specifically, IML has been shown to improve performance in the following cases:

- Small-data machine learning, where large datasets are not available, such as smartphone-based models trained on user data. Small-data machine learning requires “optimal utilisation of data” achieved by interacting with experts (or users) [56].
- Pattern mining in exploratory data analysis, which involves incrementally improving the model by incorporating user insights to identify relevant elements in the data. This approach also serves the dual purpose of enhancing model explainability through user input [56].
- Low-computing resource machine learning—improved, again, by an IML-optimised use of data [56].
- Activity recognition using a dynamic set of sensors (i.e., alternative combinations of smartphone sensors based on actual use) [57], emerged from the narrative meta-review as particularly relevant for improving the quality of passive monitoring analysis.

The last part of the strategy involves identifying when user input is critically important to improve the quality of analysis beyond baseline effectiveness. Active monitoring is typically built around structured data collection protocols, often validated to optimise the trade-off between effectiveness and user burden. A hybrid approach, however, must be designed on different premises. Instead of a pre-set data collection protocol, user prompts should be based on the confidence level of the analysis and aimed at fixing the data with a few key inputs from users.

A hidden challenge of including users in the loop is identifying information that they can assess and correct, as only a minority of smartphone sensor data falls into this category. For instance, users can correct location data to some extent but cannot evaluate the quality of heart rate measurements, step counts, or the precise time they fell asleep. *Adaptive monitoring requires rethinking the granularity level of sensor data to enable a user-informed feedback loop*. As defined, mobile biomarkers should support diagnostic tasks, such as tracking the progression of symptoms or conditions like depression, fatigue, or frailty. Low-level analyses should be abstracted away from users within models that generate high-level assessments, thereby enabling the intended user interactions (see Section 5).

### 5.3. Clinical Tasks

Diagnostic, predictive, and prognostic tasks can be seen as the clinical equivalent of pattern mining (or recognition). For instance, pattern mining for activity recognition was tested in the diagnosis of neurological conditions [58], disease progression [59], and in-hospital deaths [60]. Pattern mining involves finding sequences that lead to a similar outcome, a form of interpretation of relevant signs that can be hidden within apparently different progressions. This activity is very close to the goal of a diagnostic task, that is, finding relevant clues hidden within the data that may serve as precursors or indicators of specific conditions, thereby informing the diagnostic activity.

### 5.4. Ethics of Mobile Biomarkers

In general, integrating mHealth technologies [61] into healthcare systems can enhance their efficiency and improve outcomes in health promotion and prevention, in addition to treatment. On the one hand, the benefits of these technologies rely on lifelong, ubiquitous monitoring. On the other hand, such prolonged and pervasive monitoring raises ethical concerns, particularly regarding privacy threats. As an mHealth paradigm, mobile biomarkers fall into this category and should be the subject of careful consideration.

First of all, continuous and pervasive monitoring can be critical in most domains directly or indirectly related to health (like IoT [62]) that collect and process sensitive data. Sensitive data requires privacy-by-design solutions to protect the identity of people, who must be made aware of the modality of monitoring and data handling. The necessary informed consent can exacerbate the feeling of being surveilled and negatively affect the acceptance of mHealth and IoT solutions, even when security measures are in place. Over-surveillance must be explicitly ruled out in any protocol, along with any potential negative effects of mobile device usage on an individual’s health. Those challenges can be handled by adopting a value-sensitive design (VSD) approach [44,63]. VSD is a triadic methodology that combines conceptual (problem definition informed by disciplines such as philosophy and law), empirical (investigation of actual use informed by disciplines such as psychology and ergonomics), and technical (implementation of solutions to guide interactions, informed by disciplines such as design and engineering) studies to address the ethical challenges of technology. Mobile biomarkers present a combination of well-known challenges—-both general to mHealth and specific to digital biomarkers—that have been previously addressed in VSD studies [64,65], e.g., privacy and data security, transparency and interpretability, accessibility and ethics, and validity and reliability. Some of those criticalities are also common in all artificial intelligence (AI) domains and systems that attempt to adopt a *fair AI* approach [66], which quantifies biases and mitigates discrimination against subgroups of subjects. A fair approach could benefit any mHealth application, like mHealth digital twins (mHealth twin), built using mobile biomarker data (the term “mobile twin” has already been used in regard to twinning a mobile device [67], but with a different meaning. Hence, we use the expression “mHealth twin” [43]). However, a potential risk lies in the exploitation of mobile biomarkers in persuasive strategies for behavioural intervention, which could compromise patients’ freedom of choice. Explainable AI (XAI) solutions [68] can mitigate such risks to personal agency, making users fully aware of the information provided by the mobile biomarkers—one of the criticalities highlighted by [69] regarding the logging of life events. This would certainly increase, as discussed in similar domains, the system’s trustworthiness [70] and acceptance [71].

## 6. Conclusions

Although smartphone-only passive monitoring promises disruptive changes in public health (including AAL applications), it suffers from various technical challenges, preventing it from being accepted by patients and clinicians. In this narrative meta-review, we investigated several review papers and searched for the elements that led to the success or failure of such approaches. By conducting a SWOT analysis, we identified the *strengths*, *weaknesses*, *opportunities*, and *threats* of smartphone-only passive monitoring. In light of this analysis, we introduced the concept of *mobile biomarkers*, that is, a smartphone data analysis framework that combines multimodal summarisation and interactive monitoring. Hence, mobile biomarkers have the potential to overcome weaknesses and threats by leveraging opportunities and strengths.

The next steps in developing the concept of mobile biomarkers should address (1) use cases for data sharing and (2) the information architecture, integrating standards for medical data representation with architectures for federated and model-based machine learning. Regarding the first point, use cases should explore how users can make use of passive monitoring with third-party applications, e.g., through integration with built-in health apps (such as Google, Samsung, and Apple Health). More challenging is the sharing of data with healthcare providers in both the private and public sectors. Concerning the information architecture, the challenge is to design a solution that serves both the purposes of supporting clinical research and enabling adoption at scale. The rationale behind keeping those two activities aligned is to mitigate the risk of developing techniques that cannot be translated into actual technologies due to conflicts between research premises and real-world deployment barriers and constraints. Structurally coupling research and application would also foster a stronger partnership between smartphone makers (hardware and software) and healthcare researchers, incorporating the potentially groundbreaking benefits of passive monitoring into the design considerations of sensors and software. 

## Figures and Tables

**Table 1:** Reviews are classified according to two emerging foci, i.e., mental or physical health conditions, and the technical implementation of sensor analysis or clinical results. The purpose of this table is to provide an intuitive overview of the current landscape of available reviews.

	Mental Conditions	Physical Conditions	Technical Focus	Clinical Focus
Baxter, 2020 [12]	x	x		x
Cornet & Holden, 2018 [13]	x		x	
Higgins, 2016 [14]		x	x	
Khoo, 2021 [15]	x		x	
Krichen, 2021 [16]	x	x	x	
Kulkarni, 2021 [11]	x	x	x	
Mahsa, 2021 [7]	x		x	
Majumder & Deen, 2019 [17]		x	x	
Qirtas, 2021 [18]	x			x
Rogan, 2024 [19]	x			x
Straczkiewicz, 2021 [20]		x	x	
Stuijt, 2023 [21]	x	x		x
Zhang, 2023 [22]	x		x	
